# Cost-effectiveness analysis of the direct and indirect impact of intranasal live attenuated influenza vaccination strategies in children: alternative country profiles

**DOI:** 10.3402/jmahp.v4.31205

**Published:** 2016-06-28

**Authors:** Edward Gibson, Najida Begum, Federico Martinón-Torres, Marco Aurélio Safadi, Alfred Sackeyfio, Judith Hackett, Sankarasubramanian Rajaram

**Affiliations:** 1 Wickenstones, Oxford, UK; 2 Hospital Clínico Universitario de Santiago de Compostela, Santiago de Compostela, Spain; 3 Faculdade de Ciências Médicas da Santa Casa de São Paulo, São Paulo, Brazil; 4 AstraZeneca, Cambridge, UK; 5 AstraZeneca, Gaithersburg, MD, USA; 6 AstraZeneca, London, UK

**Keywords:** cost-effectiveness, dynamic transmission model, influenza, paediatric, LAIV, herd immunity, alternative country profiles

## Abstract

**Background:**

Influenza poses a significant burden on healthcare systems and society, with under-recognition in the paediatric population. Existing vaccination policies (largely) target the elderly and other risk groups where complications may arise.

**Objective:**

The goal of this study was to evaluate the cost-effectiveness of annual paediatric vaccination (in 2–17-year-olds) with live attenuated influenza vaccination (LAIV), as well as the protective effect on the wider population in England and Wales (base). The study aimed to demonstrate broad applications of the model in countries where epidemiological and transmission data is limited and that have sophisticated vaccination policies (Brazil, Spain, and Taiwan).

**Methods:**

The direct and indirect impact of LAIV in the paediatric cohort was simulated using an age-stratified dynamic transmission model over a 5-year time horizon of daily cycles and applying discounting of 3.5% in the base case. Pre-existing immunity structure was based on a 1-year model run. Sensitivity analyses were conducted.

**Results:**

In the base case for England and Wales, the annual paediatric strategy with LAIV was associated with improvements in influenza-related events and quality-adjusted life years (QALYs) lost, yielding an incremental cost per QALY of £6,208. The model was robust to change in the key input parameters. The probabilistic analysis demonstrated LAIV to be cost effective in more than 99% of iterations, assuming a willingness-to-pay threshold of £30,000. Incremental costs per QALY for Brazil were £2,817, and for the cases of Spain and Taiwan the proposed strategy was dominant over the current practice.

**Conclusion:**

In addition to existing policies, annual paediatric vaccination using LAIV provides a cost-effective strategy that offers direct and indirect protection in the wider community. Paediatric vaccination strategies using LAIV demonstrated clinical and economic benefits over alternative (current vaccination) strategies in England and Wales as well as Brazil, Spain, and Taiwan.

Influenza places a significant burden on healthcare systems and society. Outbreaks lead to increased mortality, reports of which range from 4.5 (in Germany, low season) to as high as 60.4 per 100,000 (in Czech Republic, high season) (1). According to a longitudinal study in the United States, 3,000 to 49,000 influenza-related deaths occur annually (2). In England and Wales, the annual burden of influenza infections has been estimated at 25,000 hospitalisations and 20,000 deaths (3). The burden of influenza in the paediatric population is under-represented (even with increasing awareness) in many childhood immunisation programmes, despite children being the major transmitters (4, 5). In young children, aged less than 5 years, and more so in those aged less than 2 years, hospitalisation rates are similar to those considered at higher risk for influenza-related complications, including the elderly population (6–8).

Limited efficacy trials with influenza vaccines (9) and historical shortage in vaccine supplies are largely responsible for prioritisation of specific groups including the elderly and high-risk groups. Recent recommendations from the Advisory Committee on Immunisation Practices (ACIP) in the United States, the Joint Committee on Vaccination and Immunisation (JCVI) in the United Kingdom, and the National Advisory Committee on Immunisation in Canada have led to administration of the influenza vaccination to all high-risk groups within the licensed indications of the available vaccines in government-sponsored programmes (2, 10, 11).

Numerous studies have modelled the clinical and economic implications of influenza strategies, including paediatric vaccination coverage (12–15). Complicating factors such as herd immunity, quality-of-life losses in young children, parental care and associated work loss, time preference, uncertainty, eradication, macroeconomics, and tiered pricing may require special consideration in economic evaluations of vaccination programmes (16). Different studies and methodological approaches address some but not all of these features; the broad consensus of these studies is that childhood vaccination is cost-effective or cost-saving (12) and should be prioritised. Infants and young children are at a higher risk of influenza-related hospitalisation and complications, and influenza is a common cause of medical office and emergency department visits in school-age children. The possibility of decreasing influenza virus transmission among children attending day-care centres and schools has been shown in economic evaluations to reduce the burden of influenza, providing both direct and indirect protection (14).

Live attenuated influenza vaccine (LAIV), a nasal vaccine first introduced to the United States in 2003, has demonstrated superior efficacy compared with inactivated influenza vaccine (IIV) in children and adolescents (12, 13, 17). Barriers to replacing IIV include licensing, which was limited to the United States until 2011, when the European Union was granted marketing authorisation (18). In 2014 ACIP gave preferential recommendation for LAIV over IIV in 2–8-year-olds (19). Pilot LAIV immunisation programmes in England targeting 4–11-year-olds during the 2013–2014 influenza season (overall uptake rate of 52.5%) imply a reduced cumulative disease incidence and swab positivity rates relative to the non-pilot and non-targeted age groups (5).

Existing vaccination models rely on extensive and detailed data on population demographics and mixing, which may be unavailable or difficult to access, particularly in the less developed world, in the depth described in Refs. (14) and (20). The objective of this study was to develop a dynamic transmission model with minimal data requirements to enable country-level exploratory modelling to assess the economic and clinical impact of implementing annual paediatric LAIV vaccination with 50% coverage in 2–17-years-olds in addition to the current vaccination policy (CVP), compared to the CVP alone for all the country settings considered (Table 1). The model operates with limited data inputs relating to country-specific epidemiology and population dynamics rather than the more complex individual patient-level data sets required by other dynamic transmission models.

**Table 1:** Model parameters, definitions, and values (base-case values) considered in transmission model for England and Wales, Spain, Brazil, and Taiwan

		Value			
		
Model parameter	Description^a^	England and Wales (base case)	Spain	Brazil	Taiwan
*t*	Time				
*P*_*T*_(*t*)	Total cohort (thousands) entering model at period *t* (47, 50, 52)	*P*_*T*_(*t*=0)=56,076 Model calculation for *t*≥1	*P*_*T*_(*t*=0)=46,592 Model calculation for *t*≥1	*P*_*T*_(*t*=0)=201,010 Model calculation for *t*≥1	*P*_*T*_(*t*=0)=23,300 Model calculation for *t*≥1
*P*_*ai*_(*t*)	Total cohort stratified by age bands at period *t*	Total cohort stratified by age bands at period *t*	Total cohort stratified by age bands at period *t*	Total cohort stratified by age bands at period *t*	Total cohort stratified by age bands at period *t*
*P*_*S*_(*t*)	Susceptible patient population at period *t*	Model calculation	Model calculation	Model calculation	Model calculation
*P*_*N*_(*t*)	Naturally immune population at period *t*	Model calculation	Model calculation	Model calculation	Model calculation
*P*_*V*_(*t*)	Effectively vaccinated population at period *t*	Model calculation	Model calculation	Model calculation	Model calculation
*P*_*Ii*_(*t*)	Infected population at period *t*	Model calculation; seed population (1,000) in Period 0	Model calculation; seed population (1,000) in Period 0	Model calculation; seed population (1,000) in Period 0	Model calculation; seed population (1,000) in Period 0
*β*	Annual birth rate,% (47, 50–52)	1.30	0.97	1.75	0.88
LE	Life expectancy, years (47, 50, 58)	85	82	74	80
CE	Clinical events,% (mortality, PCC, and hosp.) (20, 38, 48, 50)	0.13, 5.96, 0.15	0.37, 8.13, 5.65	0.42, 5.00, 1.69	1.93, 5.20, 2.27
*δ*	All-cause mortality rates,% (<2, 2–4, 5–17, 18–65, and 65 and over) (20, 38, 48, 50)	0.27, 0.01, 0.01, 0.26, and 4.95	0.16, 0.01, 0.01, 0.20, and 4.38	0.53, 0.06, 0.06, 0.36, and 4.73	0.99, 0.13, 0.09, 0.35, and 3.19
*R*_0_	Basic reproductive number (number of secondary infections originating from a single infection in *P*_*S*_) (32, 49)	1.8	1.8	1.03	1.8
*R*(*t*)	Effective reproductive number (number of secondary infections originating from a single infection at time)	Time-dependent	Time-dependent	Time-dependent	Time-dependent
*τ*(*t*)	Transmission probability of infection based on *R*.	*τ*(*t*)=1–exp(–*R*(*t*)*ρ*_*I2*_)	*τ*(*t*)=1–exp(–*R*(*t*)*ρ*_*I2*_)	*τ*(*t*)=1–exp(–*R*(*t*)*ρ*_*I2*_)	*τ*(*t*)=1–exp(–*R*(*t*)*ρ*_*I2*_)
*µ*_0_	Baseline natural immunity (assumption)	0	0	0	0
*µ*_*n*_	Duration of natural immunity (18)	Influenza A: 6 years Influenza B: 12 years	Influenza A: 6 years Influenza B: 12 years	Influenza A: 6 years Influenza B: 12 years	Influenza A: 6 years Influenza B: 12 years
*µ*_*v*_	Duration of vaccine-induced immunity for each influenza subtype (assumption)	12 months	12 months	12 months	12 months
*ω*	Coverage rate of LAIV (17)	50%	50%	50%	50%
*ρ*_*I*1_	Incubation period (32)	2 days	2 days	2 days	2 days
*ρ*_*I*2_	Infectious period (32)	2 days	2 days	2 days	2 days
*ρ*_*I*3_	Duration of infection (32)	4 days	4 days	4 days	4 days
*C*_*ab*_^b^	Contact rates between infected (*a*=1,2…5) and interaction with others (*b*=1,2…5) in the same or different age bands (<2, 2–4, 5–17, 18–64, and 65 and over) (25)	Based on the UK $$\left[\begin{array}{ccccc} 1.49 & 1.49 & 1.02 & 0.38 & 0.17\\ 1.49 & 1.49 & 1.02 & 0.36 & 0.17\\ 0.71 & 0.71 & 1.48 & 0.36 & 0.24\\ 0.40 & 0.40 & 0.39 & 0.30 & 0.16\\ 0.07 & 0.07 & 0.08 & 0.14 & 0.43 \end{array}\right]$$	Based on the Netherlands $$\left[\begin{array}{ccccc} 1.56 & 1.56 & 1.29 & 0.62 & 0.16\\ 1.56 & 1.56 & 1.29 & 0.58 & 0.16\\ 0.10 & 1.10 & 2.39 & 0.30 & 0.12\\ 0.39 & 0.39 & 0.54 & 0.41 & 0.24\\ 0.17 & 0.17 & 0.24 & 0.16 & 0.54 \end{array}\right]$$	Based on the Netherlands $$\left[\begin{array}{ccccc} 1.56 & 1.56 & 1.29 & 0.62 & 0.16\\ 1.56 & 1.56 & 1.29 & 0.58 & 0.16\\ 0.10 & 1.10 & 2.39 & 0.30 & 0.12\\ 0.39 & 0.39 & 0.54 & 0.41 & 0.24\\ 0.17 & 0.17 & 0.24 & 0.16 & 0.54 \end{array}\right]$$	Based on Poland $$\left[\begin{array}{ccccc} 1.18 & 1.18 & 0.84 & 0.21 & 0.20\\ 1.18 & 1.18 & 1.84 & 0.21 & 0.20\\ 0.69 & 1.69 & 2.64 & 0.37 & 0.26\\ 0.54 & 0.54 & 0.68 & 0.72 & 0.40\\ 0.21 & 0.21 & 0.17 & 0.25 & 0.51 \end{array}\right]$$
Coverage of CVP	CVP,% (<2, 2–4, 5–17, 18–64, and 65 and over) (35, 38, 42, 43, 46, 48, 54)	0, 2, 2, 9, and 80	0, 0, 13, 20, and 56	31, 48, 0, 87, and 80	0, 0, 0, 0, and 44
*VE*	Vaccine efficacy (74)	LAIV=80% TIV=59%	LAIV=80% TIV=59%	LAIV=80% TIV=59%	LAIV=80% TIV=59%
*V*_*S*_	Vaccination timelines,% (Jan, Feb, Mar, Apr, May, Jun, Jul, Aug, Sep, Oct, Nov, and Dec) (30, 62, 63, 78)	2, 0, 0, 0, 0, 0, 0, 0, 2, 58, 29, 9	10, 14, 1, 2, 0, 0, 0, 0, 0, 1, 7, 7	3, 7, 8, 3, 13, 18, 16, 7, 8, 14, 1, 3	16, 4, 14, 8, 12, 5, 14, 9, 4, 4, 2, 8
*Q*_*d*_	QALY decrement (45)	0.02	0.02	0.02	0.02
*C*_*E*_^b^	Costs of events (PCC, hosp., and admin.) (30, 53, 57)	£87.57 £2330.53 £35.99	£21.95 £2114.61 £21.95	£14.48 £979.22 £14.48 (same as PCC – assumption based on data availability)	£2.36 £2712.22 £2.64
*C_V_*^c^,^d^	Cost of vaccines (TIV and LAIV) (38, 41, 55–57, 59)	£5.55 £14.00	£4.02 £14.00	£2.22 £14.00	£3.55 £14.00

Admin., administration; CVP, current vaccination policy; hosp., hospitalisation; LAIV, live attenuated influenza vaccine; PCC, primacy care consultation; QALY, quality adjusted life year; TIV, trivalent influenza vaccine.

a Studies used to define the model's parameters are cited here and described in the main text. No data have been taken from one country and applied to another

b Country demographics for Spain, Taiwan, and Brazil were considered when looking for suitable contact matrices from Ref. 25 (contact rates for each age group and a selection of European countries are provided). Based on Ref. 25, the contact matrix for the Netherlands was applied to Spain and Brazil and the contact matrix from Poland was applied to Taiwan

c All costs expressed in pounds (£) with the relevant inflation (where possible) to 2014 prices (26) and currency conversions applied (62)

d The cost of LAIV in Brazil, Spain, and Taiwan is assumed to be £14.00 (the England/Wales price is used as a reference for modelling purposes).

Profiles for England and Wales (base) were explored to allow comparability with real-world and previous model outcomes and demonstrate application to other country settings. The base-case model for England and Wales takes a National Health Service (NHS) perspective to allow standardisation with published models (20) – assuming a willingness-to-pay (WTP) threshold of £30,000 in accordance to JCVI guidelines (21). To assess policy impact in countries across three different continents, profiles of Spain, Brazil, and Taiwan were explored. These countries were selected on the basis of their sophisticated vaccination calendars for influenza (Brazil, Taiwan) or, in the case of Europe, where previous models did not exist (Spain) (20, 22).

The dynamic transmission model developed simulates the expected impact of differing vaccination strategies whilst assuming population demographics differentiate between the paediatric and adult cohorts. The direct and indirect impact (herd immunity effects) of influenza transmission in a dynamic population is evaluated as well. To adapt the model to a wider range of country-specific vaccination policies, the model defines the CVP as the widely used trivalent influenza vaccine (TIV). LAIV is not under the CVP for Taiwan and Brazil. Quadrivalent influenza vaccine (QIV) is not considered in this paper because its availability is limited, particularly in the countries where the model is adapted. As per standard influenza vaccination makeup, only influenza A and B subtypes are considered because type C (the third influenza subtype) cases of influenza occur much less frequently than those of A and B (23).

## Methods

### Model structure

**Fig. 1:**
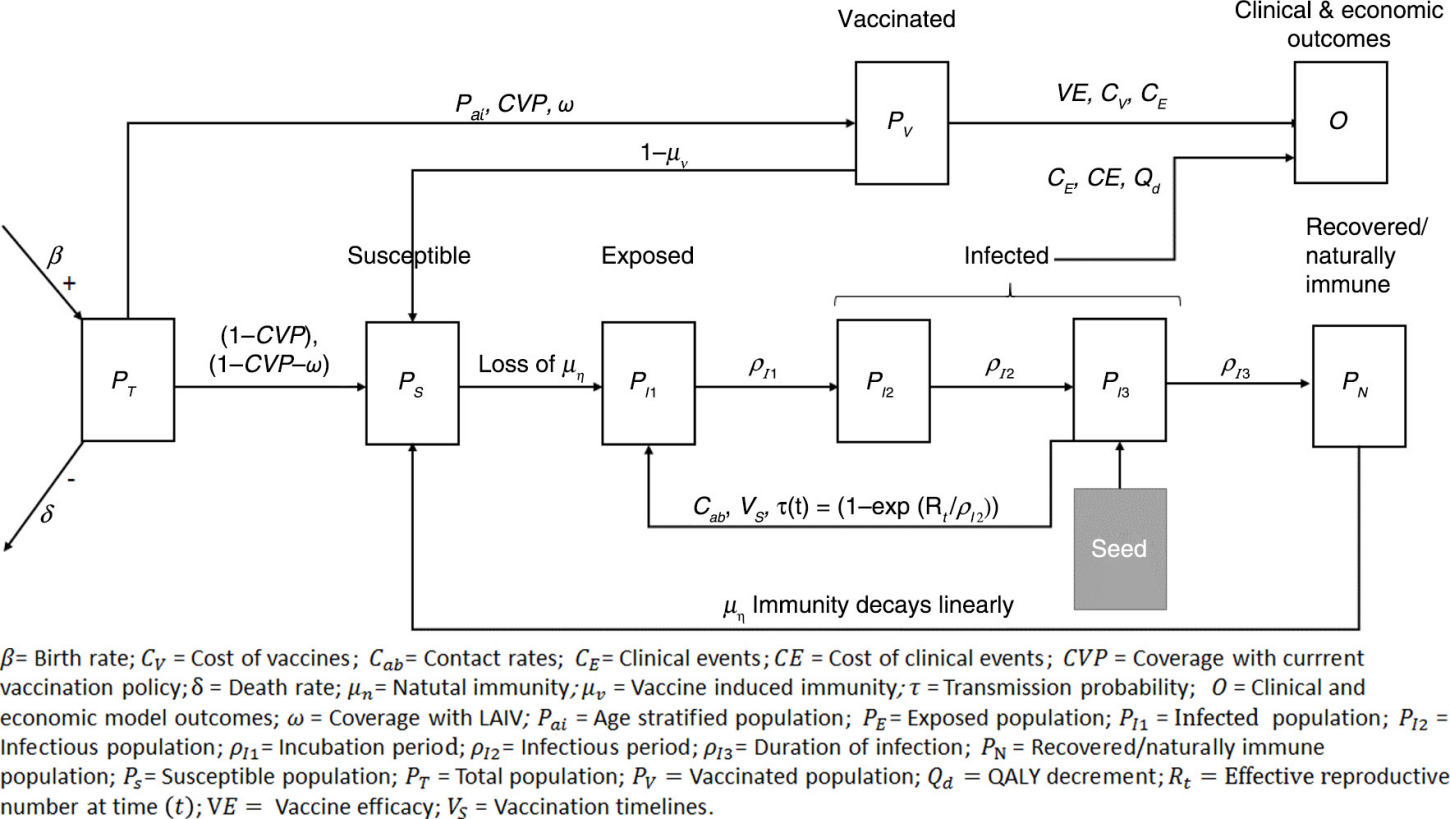
Age-stratified, dynamic, compartmentalised transmission model defines the infection and vaccination status following a simulated cohort between population compartments: susceptible (no infection or vaccination), effectively vaccinated, naturally immune (following a period of infection) and infected.

The dynamic compartmentalised transmission model developed in Microsoft Excel 2010 (Fig. 1) simulates the impact of influenza (whether symptomatic or not) and vaccination alternatives following an age-stratified population cohort *P*_*T*_(*t*)**, on a daily cycle over a 5-year horizon. The model includes waning immunity, age-specific contact rates, and seasonality of influenza transmission and follows a SEIR structure – susceptible (S), exposed (E), infected (I), recovered (R) – compartmentalising the cohort into the susceptible (*P*_*s*_(*t*)), effectively vaccinated (*P*_*v*_(*t*)), naturally immune (*P*_*N*_(*t*)), and infected populations (*P*_*I*_(*t*)=*P*_*I*1_(*t*)+*P*_*I*2_(*t*)+*P*_*I*3_(*t*)). Within these, sub-states exist and the course of infection is classified into three distinct phases: 1) *P*_1_ consists of the symptomatic (exposed) phase, which enters a latent period (*P*_*I*1_) – viral shedding does not take place; 2) infectious (*P*_*I*2_); and 3) infected and non-infectious phase (symptomatic or non-symptomatic) (*P*_*I*3_). At baseline, *P*_*I*3_ is equal to the seed population (Table 1). The seed population is used to induce initial influenza infection within the model. Full protection is assumed in those vaccinated and recovered from infection, until their immunity wanes (Fig. 1).

In the model, pre-existing immunity is generated by a 1-year model run with infection but no vaccination to generate a cohort of naturally immune individuals. The model horizon was selected to allow for at least one full cycle of natural immunity (*µ*_*n*_) and a significant period for vaccination to have an effect. Pre-populating the model with a naturally immune population is possible within the model structure to mimic a longer time horizon.

The model reports clinical and economic consequences for annual paediatric vaccination coverage with LAIV, given in addition to CVP (using TIV) *versus* CVP alone. The clinical outcomes, based on probability and cost estimates as cohort(s) transition between compartments, consider the number of primary care consultations (PCCs), influenza-related hospitalisations, and influenza-related mortality.

### Model assumptions

#### Population demographics

An age-stratified model population, $P_{T}(t)=\sum_{i=1}^{5}P_{ai}(t),$ categorises those aged less than 2 years, 2–4 years, 5–17 years, 18–64, and 65 years and over. In the model, paediatric vaccination with LAIV applies to those aged between 2 and 17 years unless otherwise stated. For a more detailed assessment of the paediatric target age groups for vaccination, 5–17-year-olds are separated into bands for 5–9 and 10–17 years (Table 3), based on a proportionate population assumption across age bands (24).

Birth rate (*β*) and population ageing are incorporated into model population dynamics. Age-related mortality (*δ*) represents the difference in mortality across the age groups – subjects of any age may experience a fatal non-influenza event, unique to each age band, and not limited to those aged 65 years and over. Influenza-related mortality is an output of the model; for simplicity, mortality is referenced to the day of infection.

#### Contact rates and infection

Transmission of influenza and exposure to infection depends on the number of contacts, proportion of infected contacts, transmission probability per contact, and interaction/mixing between the same and across different age bands; using a conservative approach, physical contact references the risk of infection. Social contacts in children and adolescents are more common when compared with other age groups. The frequency of contact defined in a European study by Mossong et al. of social contacts and mixing patterns relevant to the spread of infectious diseases (25) provides the basis for the model estimates. Assuming equal age-band distribution, the conditional mixing contact rates (*C*_*a,b*_) derived from Ref. (25) are simply averaged over these age bands (Table 1). Great Britain is used as a proxy for England and Wales.

Matching country demographics of Spain, Taiwan, and Brazil to those reported by Mossong et al. (25) – including age distribution, employment rate, and secondary education using statistics taken from the United Nations (26–29), International Labour Organisation (30), and Statistics for Development (31) – help identify a suitable contact matrix (Supplementary Appendix 2).

#### Infection, vaccination, and duration of immunity

After an initial infection, the influenza virus of both subtypes (A and B) enters an incubation period, after which infection becomes symptomatic amongst susceptible contacts (*P*_*s*_(*t*)) and they enter the exposed compartment. The basic reproductive number (*R*_0_) is defined as the number of secondary infections originating from a primary infection in *P*_*s*_. In the absence of seasonal *R*_0_ availability and consistent with previous models (20), low, moderate, and high seasons help calibrate the model such that the annual viral potency profile and within seasonal variation reflect previous influenza outbreaks occurring in 1918, 1957, and 1968 (32–34). Moderate influenza season with *R*_0_=1.8 is the base case; scenario analyses consider the low and high seasons (Table 3).

Influenza is of seasonal occurrence, with high incidence in the winter of temperate climates in the Northern Hemisphere (25). This seasonality, along with that of matched vaccination strategies (Table 1), is included in the model with variability across each month (*V*_*S*_). For the base evaluation, calculations are reliant on average monthly vaccine uptake rates (current practice) amongst general practitioner patient groups in England, in those aged under 65, 65 years and older, and pregnant women (Table 1) (35).

Risk of infection through contact (*C*_*a,b*_) is translated into a transmission probability *τ*(*t*)=1–exp(–*R*(*t*)/*ρ*_*I*2_) (25). Here, *R*(*t*) is the effective reproductive number represented as a sinusoidal function of time (generates *τ*(*t*) over the total infectious period), with maximal *R*(*t*) during January and minimal *R*(*t*) during July for the temperate Northern Hemisphere.

Infection is assumed to last 4 days based on a review from volunteer studies of infection and disease timelines in influenza (36), used in a previous model (17). Subjects (*P*_*I*1_) enter the incubation/latency period (*ρ*_*I*1_) for 2 days before they become infectious (*ρ*_*I*2_) and for a further 2 days (4). Once the infective period has passed, subjects enter a state of natural immunity (*µ*_*n*_) – reinfection with the same subtype is unlikely in the real world and, for simplicity, such an event is precluded within the model (although the model does not preclude infection with the alternative subtypes [A and B]). The number of infections determines the development of natural immunity over the course of the model. Baseline natural immunity (*µ*_0_) is assumed to be zero for all age bands. *µ*_*n*_ for influenza A and B is assumed to last 6 and 12 years, respectively (37). *µ*_*n*_ decays linearly over time to represent antigenic drift in the model.

To explore the current policy of influenza in England and Wales with TIV and the addition of LAIV to current practice, vaccine efficacy (*VE*) and the period of vaccine-induced immunity (*µ*_*v*_) are considered (38). Unlike previous publications (20), *µ*_*v*_ is conservatively assumed to be 12 months to reflect the existing annual CVP (39, 40). *VE* for LAIV and TIV are assumed to be 80 and 59%, respectively, in both paediatric and adult populations (40). The base-case coverage rate (*ω*) in the paediatric population is 50%. Coverage rates in the CVP with TIV (35) are based on estimates of total vaccinations in England mapped to the age bands of interest (a similar approach is applied to other profiles; see Supplementary Appendix 2).

#### Event rates

Risk equations are used to estimate influenza-related event rates for mortality, unplanned PCC, and hospitalisations per influenza event (Table 2) based on the results of Pitman et al.'s (20) dynamic transmission model and the findings of influenza vaccination uptake monitoring (35). Event rates per influenza infection are given by the number of each specific event (mortality, PCC, or hospitalisations) in current practice divided by the total number of influenza-related events in current practice.

**Table 2:** Clinical model outcomes for the base-case model (England and Wales), Brazil, Taiwan, and Spain of implementing an annual paediatric vaccination policy (2–17-year-olds) of LAIV in addition to current practice (assumed) with TIV

	RRR	CVP+paediatric vaccination (LAIV) (thousands)				CVP (thousands)				Δ (thousands)^a^			
				
Clinical outcome	(E&W, Brazil, Taiwan, Spain), %	E&W	Brazil	Taiwan	Spain	E&W	Brazil	Taiwan	Spain	E&W	Brazil	Taiwan	Spain
Vaccinations		21,862	160,977	4,066	19,445	15,076	132,021	1,659	16,259	7,623	28,956	2,408	3,187
Flu incidence	(19, 21, 35, 38)	6,173	18,749	4,645	6,361	9,184	30,952	5,789	8,137	(3,296)	(12,203)	(1,145)	(1,776)
Flu mortality	(6, 11, 24, 39)	5	108	103	47	6	143	110	54	(24)	(36)	(7)	(6)
PCC	(14, 17, 31, 34)	378	890	290	684	537	1,361	336	822	(173)	(470)	(46)	(138)
Hospitalisation	(19, 23, 40, 45)	17	563	246	687	27	1,040	309	899	(11)	(478)	(62)	(212)
Total costs^b^		1,018,602	3,718,627	387,967	1,276,159	644,681	3,031,080	425,178	1,334,562	373,921	687,546	37,191	(68,403)
Effects		(123)	(375)	(93)	(127)	(184)	(604)	(116)	(163)	60	244	23	35
ICER^c^										6,208	2,817	Dominant	Dominant

Annual vaccine coverage rate of 50% is assumed (negative values are given in brackets). E&W, England and Wales; CVP, current vaccination policy; ICER, incremental cost-effectiveness ratio; LAIV, live attenuated influenza vaccine; PCC, primary care consultations; QALY, quality adjusted life years; TIV, trivalent influenza vaccine; RRR, relative risk reduction.

a Difference in costs divided by the difference in QALYs

b costs include the avoidance of influenza events. Discount rates of 3.5% for E&W and 3.0% for Brazil, Taiwan and Spain were applied to model a horizon of 5 years (including a 1-year model run)

c all model outcomes have been rounded to the nearest whole number. Calculations based on the tabulated values will differ slightly due to rounding simplifications. Dominant favours the intervention (fewer costs and more effects are observed).

#### Resource use and costs

The cost of TIV was taken as the mean price for nine IIVs from the British National Formulary (BNF, 67 March 2015) – £5.55. The cost of LAIV is assumed for modelling purposes to be £14.00 (41).

In addition to the vaccine itself, an administrative cost (consultation and dispensing fee) is assumed. The assumed setting (primary care) represents a resource-intensive approach to vaccination and alternative, less costly approaches, which may be facilitated by nasal administration, such as clinic-based vaccination or vaccination on site in schools, are being adopted in the real world. Conservatively, costs are assumed equivalent for administration of both TIV and LAIV, although in reality the administration of LAIV may offer a cost-saving potential with minimal administrative costs based on recommendations to allow healthcare assistants to take over nursing duties (42) (Table 1).

Costs for unplanned PCC – including the cost of antibiotics, antiviral drugs, and complications – and hospitalisations, including hospital attendance and pneumonia episodes, for influenza-related episodes were accounted for; costs consider the total number of influenza-like illness (ILI) consultations stratified into staff salary and prescription costs, as well as antibiotics treatment during ILIs taken from the Clinical Practice Research Datalink (CPRD) between 2000 and 2009 (43) and expressed as 2015 price levels using the Hospital and Community Services Price and Pay Index (44) (Table 2).

All events and costs are discounted within the model at a rate of 3.5% in the base case (England and Wales) and 3.0% for the other countries (20).

#### Quality-adjusted life years

In the absence of data for the paediatric population, but consistent with previous model assumptions (20), a quality-adjusted life year (QALY) decrement of 0.02 (applies to –*P*_*I*_(*t*) within the infectious period (*ρ*_*I*2_), based on a 21-day duration of an influenza event from Turner et al.'s economic decision modelling for the prevention and treatment of influenza A and B (45)), is assumed for all age bands. Limitations in the paediatric population are evident in other studies with adult QALY/utility used to populate models and without distinction between hospitalised and non-hospitalised populations (20, 46).

Life years lost are calculated assuming mortality is evenly distributed within an age band and compared with remaining life expectancy for the general population. In the base case, a life expectancy of 85 years is assumed (47).

### Alternative country profiles

The core model framework has extensive capabilities to be adapted to alternative country profiles, where access to data is minimal. Model input parameters for the *C*_*a,b*_, *VE*, *µ*_*n*_, *µ*_*v*_, *ω*, and QALY remain unchanged with alternative country profiles. For Brazil, Spain, and Taiwan, data for country demographics (population distribution, life expectancy, birth rates, and mortality rates), *R*_0_ (base-case value applied in the absence of suitable data), CVP strategy, vaccination timelines, event rates, and costs were sourced from the literature (details presented in Supplementary Appendix 2) (48–65). All costs were inflated, where possible, and expressed in 2015 prices using the Consumer Price Index and foreign exchange rates (May and July 2014) (66–68), with the exception of cost of complications where costings form Personal social services research unit (PSSRU) 2015 were taken (Table 1).

### Analyses

Costs and QALYs were calculated over the model horizon and presented as a mean outcome per year. The estimated costs and QALYs based on pairwise differences provide the incremental cost-effectiveness ratio (ICER).

Although probabilistic sensitivity analyses (PSAs) are not recommended in dynamic models (69), they have been used previously (20) in the cost-effectiveness analysis of LAIV. Here, the PSA applies Monte Carlo simulation to propagate uncertainty in the estimated ICERs by randomly sampling model input parameters simultaneously for 1,000 iterations. All inputs were varied according to the same distributions as one-way sensitivity analyses. Univariate sensitivity analyses investigate the individual impact of the upper and lower limits for the key model parameters. All sensitivity analyses consider a 95% confidence interval for the standard normal distribution (where possible) (70, 71) or a 25% uncertainty margin is applied to the mean input parameter values (Fig. 3). A WTP threshold of £30,000 is assumed for the PSA in the base-case model (Fig. 4a and b) (72). Further scenario analyses conducted assess model outcomes to include change in paediatric age bands, choice of vaccine, *R*_0_, and *µ*_*n*_ (base-case setting: Table 3). Alternative country profiles are also considered.

**Table 3:** Cost-effectiveness analyses comparing CVP with selected paediatric vaccination strategies (ages 2–4, 2–10, and 2–17) of LAIV or TIV in addition to current practice and scenario analysis conducted on the base-case model

	Costs (£, thousands)^a^			Effects (thousands)			ICER (£)
			
	CVP+paediatric vaccination	CVP	Δ costs	CVP+paediatric vaccination	CVP	Δ QALYs	(ΔC/ΔQ)^b^
Current vaccination strategies							
CVP+LAIV (2–4 years) *vs*. CVP	767,594	644,681	122,913	(175)	(184)	9	13,671
CVP+LAIV (2–10 years) *vs*. CVP	856,938	644,681	212,257	(152)	(184)	32	6,733
Base case (Table 2)	1,018,602	644,681	373,921	(123)	(184)	60	6,208
CVP+TIV (ages 2–4) *vs*. CVP	742,604	644,681	97,923	(160)	(163)	3	13,268
CVP+TIV (ages 2–10) *vs*. CVP	817,594	644,681	172,913	(145)	(128)	18	7,290
CVP+TIV (ages 2–17) *vs*. CVP	952,864	644,681	308,184	(126)	(163)	37	6,898
CVP+LAIV (ages 2–17) *vs*. CVP+TIV (ages 2–17)	1,018,602	952,864	65,737	(112)	(126)	13	4,226
Scenario analyses (base-case model)							
Low season *R*_0_=1.08	1,007,113	632,107	375,006	(91)	(148)	57	6,586
High season *R*_0_=3.6	1,027,975	655,098	372,877	(149)	(214)	64	5,791
*µ*_*n*_ – Influenza A (2 years) Influenza B (12 years)	1,036,821	678,353	358,467	(181)	(297)	115	3,096
Price parity (LAIV=TIV)	948,019	644,681	303,339	(123)	(184)	51	5,036
Cost per vaccine – admin. (LAIV=£0)	228,120	132,968	95,152	(123)	(184)	95	1,580
Efficacy (LAIV=TIV)	1,023,445	644,681	378,765	(139)	(184)	37	8,477

An uptake rate of 50% is assumed. Admin., administration; CVP, current vaccination policy; ICER, incremental cost-effectiveness ratio; LAIV, live attenuated influenza vaccine; QALY, quality adjusted life years; TIV, trivalent influenza vaccine

a Discount rates of 3.5% were applied over the 5 year model horizon (including a 1-year model run)

b Incremental costs divided by incremental QALYs.

## Results

### Base case analysis

The addition of LAIV (2–17-year-olds) to the CVP (CVP +LAIV) *versus* CVP alone formulates the base-case scenario (Table 1 provides all model parameter values).

**Fig. 2:**
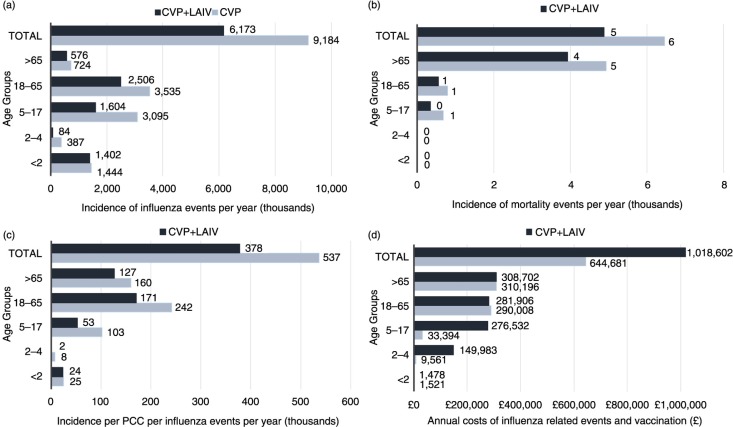
Base-case results per age band for CVP+LAIV and CVP alone for (a) incidence of influenza events per year; (b) incidence of influenza mortality events per year; (c) incidence of PCC per influenza event per year; and (d) total costs per year (all values given to the nearest whole number). All projections were benchmarked to existing (pre-paediatric vaccination) values. CVP, current vaccination policy; LAIV, live attenuated influenza vaccine; PCC, primary care consultations.

Clinical model outcomes demonstrate fewer influenza events (relative risk reduction [RRR]=36%), influenza-related mortality (RRR=40%), PCC (RRR=32%) and hospitalisation (RRR=41%) with CVP+LAIV (Table 2). Further breakdown per age band (Fig. 2a) illustrates an increased number of influenza events particularly in those aged 18–64 under CVP alone – highlighting a need for paediatric vaccination to provide indirect protection in the wider community. PCC and mortality events (Fig. 2b and c) are greater in the ‘at risk’ elderly population (65 years and over) and those aged 18–64 years, respectively, with CVP alone; this figure is also supported by the total costs accrued in this age band (Fig. 2d).

The model policy (CVP+LAIV) incurs an increased number of vaccinations (Table 3) and has an impact on associated costs (38%) supported by Fig. 2d. The benefits of annual paediatric vaccination improve both the number of life years (31%) and the QALYs lost (33%). Comparisons with CVP alone per age band (Fig. 2a) support the loss of QALYs projecting in the adult population (higher number of infections) – particularly for 18–64-year-olds.

Paediatric vaccination with CVP+LAIV *versus* CVP alone was associated with an incremental cost of £373,921,000 and QALY gain of 60,236, yielding a cost per QALY of £6,208, which can be considered cost-effective assuming a WTP threshold of £30,000. CVP+LAIV is also associated with 51,000 life years gained (LYG) and 171,000 influenza events averted, giving a cost per LYG of £7,374 and savings of £2,186 for each influenza event averted per year.

### Sensitivity analyses

**Fig. 3:**
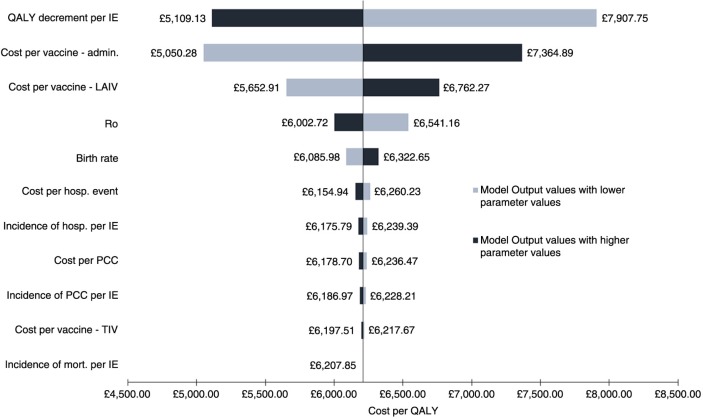
Base-case results for the univariate sensitivity analysis of the incremental cost-effectiveness ratio. The range was produced by applying 95% confidence interval where possible or a 25% uncertainty margin to the mean input parameter values (see Supplementary Table 4). Admin., administration; hosp., hospitalisation; ICER, incremental cost-effectiveness ratio; IE, influenza event; LAIV, live attenuated influenza vaccine; mort., mortality; PCC, primary care consultation; QALY, quality adjusted life years; TIV, trivalent influenza vaccine.

Univariate sensitivity analysis conducted on key model parameters implies that the model is robust to plausible changes (Figure 3, with many having minimal impact on the overall ICER). The model was sensitive to changes in the QALY decrement, administrative costs (vaccination-related), and cost of LAIV. The QALY decrement is a key driver where substantial differences (RRR=32%) in the incidence of influenza events and vaccination coverage between CVP+LAIV and CVP alone – noticeable in the paediatric age bands and those aged 18–64 years (Fig. 2a) – contribute to the loss of QALY under CVP alone. The administration costs of vaccination are another driver, as expected, with 50% coverage in the paediatric population, leading to a significant increase in costs (38%) between CVP+LAIV and CVP alone. Unsurprisingly, the cost of LAIV has an impact on the overall ICER with an approximate threefold price difference compared with TIV.

**Fig. 4:**
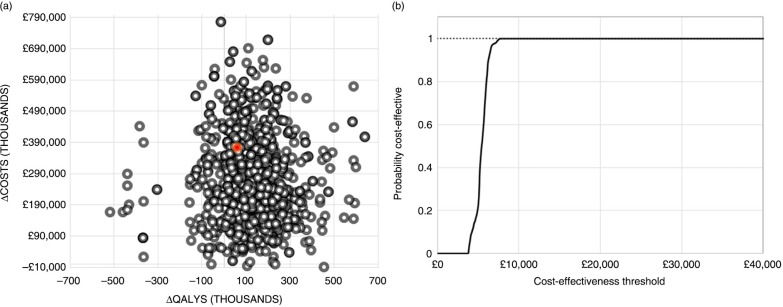
(a) Base-case scatterplot generated by applying a normal distribution in the PSA; (b) CEAC generated from the PSA. CEAC, cost-effectiveness acceptability curve; ICER, incremental cost-effectiveness ratio; PSA, probabilistic sensitivity analysis; QALY, quality adjusted life years.

Uncertainty generated from simulations of the PSA is largely associated with a reduction in QALYs lost due to influenza at an incremental cost. The probability of CVP+LAIV being cost-effective for the WTP threshold of £30,000 was greater than 99% (Fig. 4).

### Scenario analyses

Extending annual paediatric vaccine coverage with LAIV (or TIV) in addition to the CVP *versus* CVP alone for those aged 2–4, 2–9, and 2–17 was compared (Table 3). Vaccine coverage with LAIV in 2–4-year-olds provided a cost-effective strategy, resulting in an ICER of £13,671 (TIV: £13,268). Expanding coverage in 2–9-year-olds improved the ICER to £6,733 with LAIV (50%) and ICER of £7,290 with TIV (45%). Further expansion to the full paediatric population resulted in an ICER of £6,208 (base case) with LAIV (55 and 8% reduction compared with 2–4 and 2–9-year-olds, respectively), and the addition of TIV provided an ICER of £6,898 (48 and 5% reduction compared with 2–4 and 2–9-year-olds, respectively). Additionally, full paediatric coverage using CVP+LAIV *versus* CVP+TIV remains cost-effective with an ICER of £4,226. Overall, extending annual paediatric immunisation in addition to CVP provides cost-effective strategies with the greatest benefits presented in those aged between 2 and 17 years (CVP+LAIV). The comparisons between LAIV and TIV are driven by the significant cost differences between the two vaccines; LAIV is responsible for better improvements with extended coverage (2–4 and 2–17-year-olds). With price parity (Table 3) an ICER of £5,036 is obtained.

Reducing administrative costs with LAIV (Table 3) impacts on the overall ICER (£1,580). Although a real cost associated with administration still applies, this highlights the cost-saving potential reflective of healthcare assistants taking over tasks from nurses in the future with LAIV (38). The intranasal method may also be better tolerated in the paediatric population than injectable vaccines and provides a highly cost-effective alternative. Reducing the duration of natural immunity for influenza A, the most virulent subtype, yields an ICER of £3,096. This may mimic an antigenic shift where an abrupt change in the virus can result in a loss of immunity from previous infections and updated or regular vaccinations will benefit the wider population.

The ICER remained cost-effective for extreme values for *R*_0_ (*R*_0_=3.6–£5,791 and *R*_0_=1.08 – £6,586) and when the same efficacy for both LAIV and TIV was assumed (£8,477). This finding could imply fewer secondary cases with CVP+LAIV in the wider population.

### Alternative country profiles (Brazil, Taiwan, and Spain)

The implementation of annual paediatric vaccination with CVP+LAIV in Brazil, Spain, and Taiwan provides effective strategies when compared with the CVP alone. Based on the model outcomes, fewer influenza events (Table 3), related mortality, hospitalisations, and PCC occurred in all three country profiles – consistent with the base-case results. In Brazil an incremental cost of £687,546,000 and a QALY gain of 244,060 yields an ICER of £2,817. In Spain, the incremental cost of £68,403,000 and a QALY gain of 35,583 provide a dominant ICER. In Taiwan, an incremental cost of £37,191,000 and a QALY gain of 22,892 give a dominant ICER.

The selected profiles differ in terms of existing vaccination coverage and event rates that exert an impact on the overall ICER. The CVP in Spain and Brazil includes the paediatric population (Table 1), although this population seems absent in Taiwan (based on model inputs). Event rates for PCC, hospitalisation, and mortality vary, with greater hospitalisations reported in Spain (Table 2). Sensitivity analyses are presented in Supplementary Appendix 2; QALY decrement per influenza event, cost of vaccine (LAIV), and administration of vaccine contribute to the overall ICER.

## Discussion

Previous economic evaluations (15, 20) investigated the implications of adding paediatric vaccination strategies to the CVP that largely target elderly and other at-risk groups. These analyses all suggest that employing annual paediatric vaccination will provide a more effective and cost-effective strategy than the existing CVP in place. The complexities seen in previous models (13, 17) and the extensive data needed to populate these models influenced the development of the dynamic transmission model presented here, with minimal data requirements to enable country-level exploratory modelling. The potential impact of annual paediatric vaccination with LAIV was explored, capturing the clinical, economic, and cost consequences for influenza events based on PCC, hospitalisation, related mortality, and QALYs lost.

The base-case analysis for England and Wales indicates that, consistent with previous models, annual paediatric vaccination (CVP+LAIV) in those aged 2–17 years provides a more cost-effective strategy than CVP alone (with lower paediatric coverage). The results assume a WTP threshold of £30,000 and a 50% coverage. The incremental cost was £373,921,000 and the incremental QALYs lost was 60,236, yielding an ICER of £6,208. The model supports findings from the pilot programmes in England, with LAIV presenting an overall reduction in incidence for a wide range of influenza indicators, including PCC and hospitalisation (5).

Change in key model parameters (Figure 3) had minimal impact on the overall ICER. Sensitivity to change in administrative costs, QALY decrement, and cost of LAIV was observed. Results from the PSA imply that annual paediatric vaccination improves the QALYs lost at an incremental cost. The probability of CVP+LAIV being cost-effective was more than 99%, based on the WTP.

Scenario analyses (Table 3) demonstrated that adding 50% coverage with either TIV or LAIV to CVP was more effective than CVP alone (with low paediatric coverage), although LAIV was more efficacious and may offer a cost-saving potential with nasal administration. The findings also suggested that vaccination in the full paediatric population (2–17 years) *versus* vaccination in selected paediatric groups (e.g., 2–4) was more effective. Annual paediatric vaccination provides direct and indirect protection (herd immunity) from influenza infections and related events (73). Children are major transmitters in the wider community and their interactive role with those who do not fall into the CVP will result in their indirect protection. This may include the unvaccinated or other at-risk groups, such as those with comorbidities or pregnant women, in the same or different age bands. Additionally, vaccination strategies for varying levels of coverage (Table 3) are in alignment with scenarios presented by Pitman et al. (20). Consistent with previous models, each strategy is cost-effective by conventional thresholds. Variations in absolute values for ICER may be driven in part by the simplified model structure (shortened time horizon – 4 years *vs*. 200 years, age banding *vs*. individual year cohorts) and in part by alternative input assumptions such as duration of immunity for vaccine being defined independently of natural immunity in the current model (20).

Adapting this model to alternative country profiles for Brazil, Spain, and Taiwan captures population demographics, vaccination strategy, seasonality, and costs for each profile (other parameters can be updated if available) to provide the clinical and economic outcomes seen in the base model. All profiles benefit from annual paediatric vaccination with LAIV. The model flexibility is particularly useful for countries with limited epidemiological and transmission data.

Several improvements from previous models have been made. In the model, immunity develops following an influenza infection. The duration of natural immunity and vaccine-induced immunity differ. Vaccine-induced immunity is conservatively assumed to last 12 months for both subtypes. The model is based on a short time horizon, capable of simulating the effects of influenza infection similarly to those seen in the dynamic transmission model of Pitman et al. (20).

Models of dynamic transmission of influenza are subject to intrinsic limitations due to their inability to appropriately track susceptible population contact with infectious individuals and to appropriately estimate basic reproductive number and its seasonal variation, together with the recognition of the impact of herd immunity (16).

The current study has limitations and provides a simplification of what is expected in reality to simulate the viral spread of influenza. Long-term outcomes, believed to reflect the consequences of annual paediatric vaccination, were based on a number of assumptions about contact rates and mortality (discussed below) in order to capture natural disease progression.

The purpose of the model is to highlight the benefit of annual vaccination in the full paediatric population stratified into age bands, in a similar way to Pitman et al.'s model (20), rather than using precise age cohorts (e.g., 0, 1, 2, 3, …, 100). Information presented by Mossong et al. (25) considering social contacts and mixing patterns relevant to the spread of infectious diseases provides the basis for the contact rates applied to the model. Contact rates derived from Mossong et al. (25) are used to estimate the relevant cross-age-dependent rates – by simply taking the averages, rather than the complex dual-weighted average for the contactor and contactee. This may over- or underestimate some effects of individuals becoming infected, but has a minor effect in the context of the uncertainties around other inputs. Additionally, matrices selected by matching country demographics for Brazil, Spain, and Taiwan may not be truly reflective of contact patterns across the age groups.

It is assumed that birth and mortality rates are maintained throughout the period of the model and have been largely consistent over the preceding periods. These simplifications may have an impact on the model in terms of transition probabilities between age bands.

Many of the inputs to the model remain uncertain and the best available data have been used to drive these. For example, the use of contact matrices may be skewed due to selection bias in the original studies. The QALY decrement is uniform across all age bands and provides no differentiation between the paediatric and adult populations or between hospitalised and non-hospitalised infected populations. In the absence of suitable data, this approach is also adopted in other economic models (17, 42), although the decrement in the paediatric population may be greater than that in the other age bands and is not fully captured with the current QALY decrement applied. Based on data availability, the model applies normal distributions to each parameter value in the probabilistic analyses.

The natural history and dynamics of influenza infection are assumed throughout the model to be constant irrespective of both the age of the individual infected and the strain of virus. There is some evidence to suggest that the natural history of influenza is to some extent influenced by the age of the host. Good data on these differences are sparse, however.

The model also assumes that infection with influenza leads directly to natural immunity. This overemphasises the protective effect of infection compared with estimates in the literature (74). This conservative assumption reduces the potential benefit of vaccination in comparison with the natural course of disease.

The fixed time horizon for both natural and vaccine-induced immunity to influenza infection is a necessary simplification within the model. This does simplify the overall flow of patients, however, and may exaggerate or dampen the effects of both vaccination and natural immunity.

Comparative analyses with QIV were not considered due to composition of the vaccine with both influenza B lineages, for which the benefits can only be assessed over a longer time horizon, which was beyond the scope of the current model framework and time horizon. With flexibility in the model framework, future scope could extend the current analysis to capture the impact of QIV with some minor amendments.

Finally, the costs of implementing a vaccination policy are assumed to be fixed and, conservatively, to occur within the most expensive setting. It is likely that in reality vaccination costs vary depending on the individual/implementation methods. For instance, school-based programmes are increasingly being adopted and thus reducing costs (75), whereas harder-to-reach populations require significant investment, thus raising the cost. In contrast, other individuals are vaccinated at very low costs in clinic or outreach settings.

## Conclusions

The model provides a facile and relatively low data requirement methodology for assessing the impact of the introduction of paediatric vaccination programmes for influenza and yields results that are consistent with previous, high-data models and real world evidence from pilot programmes.

Overall, annual paediatric vaccination with LAIV in addition to current practice yields an ICER of £6,208 (base-case model). The core model framework can be used for alternative country profiles with minimal data and limited adaptation, as shown here for Spain, Taiwan, and Brazil, demonstrating the clinical and economic benefits of paediatric vaccination strategies using LAIV over alternative strategies.

The model highlights a need for wider coverage in the paediatric population, where indirect protection arising from herd immunity may make a significant contribution to the effectiveness of overall vaccination strategies.

## Supplementary Material

Cost-effectiveness analysis of the direct and indirect impact of intranasal live attenuated influenza vaccination strategies in children: alternative country profiles

## Supplementary materials

Details of the systematic review conducted to inform the model design, and specify input parameters are given in S1. The model input parameters for the alternative country profiles and univariate sensitivity analyses for Spain, Taiwan and Brazil are detailed in S2.
