# Supplementary material for: Cost-effectiveness analysis of the direct and indirect impact of intranasal live attenuated influenza vaccination strategies in children: alternative country profiles
Source: J Mark Access Health Policy. 2016 Jun 28;4:10.3402/jmahp.v4.31205. doi: 10.3402/jmahp.v4.31205 (PMC4928186; doi:10.3402/jmahp.v4.31205)
Supplement: Cost-effectiveness analysis of the direct and indirect impact of intranasal live attenuated influenza vaccination strategies in children: alternative country profiles [file JMAHP-4-31205-s001.doc]

Supplementary Appendix 1: Systematic review

Accompanying manuscript:

# “Cost-effectiveness analysis of the direct and indirect impact of intranasal live attenuated influenza vaccination strategies in children: alternative country profiles”

# Introduction

To support, inform and populate the influenza transmission and vaccination model and specify model inputs– systematic reviews in PubMed (Medline), The Cochrane Library and the Google Scholar were performed. The search accounted for population demographics, existing health economic studies (costs and utility measures), vaccine efficacy, vaccination policies, spread of disease, seasonality, reviews/national reports, information for the reproduction number and social contacts/mixing patterns between age bands within the influenza indication for the target population (including the paediatric and elderly populations). The data extracted was assessed for its suitability first to the core model framework and then the alternative country profiles of interest (with scope to capture profiles not currently defined).

**Table S1. Model parameter groups and information expected from the literature search.**

| **Category** | **Information expected** |
| --- | --- |
| **Population** | Country demographic data from statistical databases |
| **Birth rate** | Country demographic data from statistical databases |
| **Mortality** | Country demographic data from statistical databases |
| **Life expectancy** | Country demographic data from statistical databases |
| **Contact matrix** | Spread of infection through social contacts |
| **Reproduction number** | Transmission of infection |
| **Natural Immunity** | Baseline immunity |
| **Infection** | Duration of each infective period |
| **Vaccination strategy** | Existing strategy in place – coverage rates unique to age bands |
| **Vaccination effectiveness** | Vaccine types and associated efficacy |
| **Duration of natural immunity** | Protection from influenza following infection/exposure |
| **Duration of vaccine induced immunity** | Protection from influenza |
| **Vaccination timeline** | Seasonality |
| **QALY** | The impact on a patient’s quality of life (QoL) |
| **Event rate** | The number of influenza related events |
| **Costs** | Costs of events and vaccinations |

# Literature databases

- PubMed search considered articles from 1^st^ Jan 1980 to 14^th^ May 2014
- The Cochrane library search considered articles from 1^st^ Jan 1999 to 14^th^ May 2014

The search was limited to articles published in English and human studies.

# Internet sites and other sources

- The Google scholar search was conducted between 7-14^th^ May 2014, to identify recent abstracts, publications, health technology appraisals and relevant documents with no limits on the publication date.

# Search terms

For the electronic databases the search terms included combinations of title/abstract free-text terms and subject index heading (e.g. medical subject headings [MeSH]).

The key components captured in each category are summarised below.

- Indication: Influenza, subtypes, immunization and vaccines.
- Models:
  - Cost-effectiveness models, other cost models (cost-benefit, cost of illness and cost-burden), dynamic transmission model, decision models, economic evaluations/analysis.
  - Quality of life related studies (utility measures, quality adjusted life years, acceptability and health impact).
- Spread of disease:
  - Spread of disease (epidemic/pandemic, outbreaks and global burden).
  - Immunity.
  - Vaccination policy (programmes/schedules/strategies and prevention/control).
  - Vaccine uptake rates/monitoring/impact/transmissibility/efficacy.
  - Infections (type, seasonality, hospitalisation, referrals and consultations).
- Reports/reviews: Systematic reviews, public health reports and patient related outcomes.
- Mortality: Related/cause of death.
- Target population: Paediatric and elderly populations.
- Parameters: Reproduction number, transmission parameters, mixing patterns/social contacts.

# Inclusion and exclusion criteria

All articles retrieved from the literature review were screened first by title, second by abstract and finally by reading the remaining full text articles. Results are reported according to the Preferred Reporting Items for Systematic Reviews and Meta-Analysis [PRISMA].

Articles were excluded if they considered (1) indications, outcomes or studies non-influenza related; (2) specific subgroups as this was not the focus of the paper e.g. flu and asthma or cancer and flu; (3) animal studies; (4) non-English articles

**Table S2.** Search strategy terms compiled using PubMed [MEDLINE] (search timeline: 1980 – 14 May 2014)

| **Category** | **Search number** | **Search terms** | **#items** |
| --- | --- | --- | --- |
| **Indication** | #1 | (((((((((((((((((("influenza vaccines"[mh]) OR "influenza"[tiab]) OR "h1n1"[tiab]) OR "h5n1"[tiab]) OR "h3n1"[tiab]) OR "haemophilus influenza"[tiab]) OR "influenza like illness"[tiab]) OR "ILI") OR "avian influenza"[tiab]) OR ("influenza vaccines/administration and dosage"[mh])) OR "influenza vaccine uptake") OR "attenuated influenza") OR "live attenuated influenza vaccine") OR "intranasal"[tiab]) OR "hib") OR "laiv") OR "flumist") OR "fluenz") OR "influenza vaccinations"[tiab]) OR "influenza epidemic") OR "influenza epidemic outbreaks") OR "influenza a/b") OR "flu") OR "flu vaccine") OR "seasonal flu vaccine") OR "seasonal influenza epidemic") OR "seasonal influenza vaccines") OR "influenza immunisation") OR "influenza immunization"))))))) | 92,754 |
| **Models** | #2 | ((("cost effectiveness"[tiab]) OR "cost-effectiveness"[tiab]) OR "cost-effectiveness analysis"[tiab]) OR "cost-effectiveness model"[tiab]) OR "cost effectiveness model"[tiab]) OR "cost-benefit model"[tiab]) "cost benefit model"[tiab]) OR "cost-benefit analysis"[tiab]) OR "cost burden"[tiab]) OR "cost-burden"[tiab]) OR "cost of illness"[tiab]) OR "cost-burden"[tiab]) OR "cost-analysis"[tiab]) OR "cost analysis"[tiab]) OR "dynamic transmission model"[tiab]) OR "transmission model"[tiab]) OR "dynamic transmission"[tiab]) OR "effectiveness"[tiab]) OR "economic decision modelling"[tiab]) OR "decision model*"[tiab]) OR "decision") OR "economic evaluations"[tiab]) OR "economic analysis"[tiab]) OR ("benefits and costs"[tiab]) OR "SEIRS model"[tiab]) OR "susceptible exposed infected resistant") OR ((("quality of life") OR "quality of life/health related quality of life hrqol") OR "qaly") OR "quality of health care/economics") OR "qaly/gained") OR "utility/benefit") OR "utility/acceptability") OR "utility/cost") OR "utility/cost effectiveness") OR "utili*"[tiab]) "health impact"[tiab]) OR "healthcare resource"[tiab]) OR "productivity"[tiab]) OR "socio-economic"[tiab]) OR "socioeconomic"[tiab]))) | 582,989 |
| **Spread of disease** | #3 | ((((((((("spread of disease"[tiab]) OR "global burden"[tiab]) OR "world health") OR "infections"[tiab]) or "infectious"[tiab]) OR "disease"[tiab]) OR "prevention"[tiab]) OR "control"[tiab]) OR "treatment"[tiab]) OR "pandemic"[tiab]) OR "pandemic") OR "epidemic") OR "epidemic"[tiab]) OR "risk factors"[tiab])OR "disease outbreaks*") OR "programmes"[tiab]) OR "programmes") OR "schedule"[tiab]) OR "schedule") OR "program*"[tiab]) OR "vaccination program*"[tiab]) OR "state medicine"[mh]) OR "health policy"[tiab]) OR "strategy"[tiab]) OR "strateg*") OR "health"[tiab]) OR "efficacy") OR "efficacy"[tiab]) OR "dose"[tiab]) OR "dosage"[tiab]) OR "hospitalisation"[tiab]) OR "hospitalization"[tiab]) OR "hospitalization"[mh]) OR "hospitali*") OR "disease control") OR "disease control and prevention") OR "control") OR "disease prevention") OR "centers for disease") OR "centres for disease") OR "vaccine uptake monitoring") OR "respiratory infections"[tiab]) OR "respiratory tract infections"[tiab]) OR "seasonality"[tiab]) OR "respiratory"[tiab]) OR "pneumococcal"[tiab]) OR "pueumonia"[tiab]) "epidemic control"[tiab]) OR "epidemic prevent*") OR "referral") OR "consultation") OR "vaccine immunity") OR "vaccine immunity"[tiab]) OR "vaccine induced immunity") OR "vaccine induced immunity"[tiab]) OR "natural immunity") OR "natural immunity"[tiab]) OR "vaccine effectiveness") OR "vaccine effectiveness"[tiab]) OR "duration of immunity") OR "immunity timeline") OR "annual"[tiab]) OR herd immunity[mh]) "annual production cycle"[tiab]) OR vaccination policy[mh]) OR "optimal target populations") OR "transmissibility"[tiab]) OR "epidemiological impact"[tiab]) OR "epidemiolo*"[tiab]))))) | 3,404 |
| **Reviews/reports** | #4 | ((("systematic reviews"[tiab]) OR "systematic review"[tiab]) OR "target populations"[tiab]) OR "systematic analysis"[tiab]) OR "literature review"[tiab]) OR "literature"[tiab]) OR "department of health") OR "department of health"[tiab]) OR "public health") OR "public health"[tiab]) OR ("modelling study"[tiab]) OR "model*"[tiab]) OR "studies"[tiab]) OR "patient-relevant clinical trial outcomes"[tiab]))) | 4,020,746 |
| **Mortality** | #5 | ("mortality"[tiab]) OR "mortality") OR "death") OR "death"[tiab]) OR "cause of death"[tiab]) OR "fatality") OR "fatality"[tiab]) OR "related death"[tiab]) OR "related-death"[tiab]) "fatal cases"[tiab]) | 1,356 |
| **Parameters** | #6 | (("estimating"[tiab]) OR "estimation"[tiab]) OR "reproduction number"[tiab]) OR "reproductive number"[tiab]) OR "R0"[tiab]) OR "basic reproduction number"[tiab]) OR "transmission parameters"[tiab] OR "transmission") OR "mixing patterns") OR "mixing patterns"[tiab]) OR "patterns"[tiab]) OR "social contact"[tiab])) | 536,275 |
| **Target population** | #7 | (("paediatric") OR "pediatric") OR "adolescent") OR "adult" OR "young adulthood") OR ("aged") OR ("aged, 80 and over")) OR ("65 and over") OR "child") OR "children") OR "infant") OR "under 18") OR "newborn")) | 7686573 |
| **Exclusion criteria** | #8 | (((((((((("pregnancy"[ti] OR "diabetic"[ti] OR "HIV"[ti] OR "renal"[ti] OR "diabetes"[ti] OR "cancer"[ti] OR "meningitis"[ti] OR "methicillin-resistant Staphylococcus aureus"[ti] OR “MRSA”[ti] OR "Bifidobacterium"[ti] OR "myocarditis"[ti] OR "syncytial virus"[ti] OR "pregnant"[ti] OR "microbial exposure"[ti] OR "brucellosis"[ti] OR "splenectomy"[ti] OR "heart disease"[ti] OR "bronchiolitis"[ti] OR "myeloid leukemia"[ti] OR "carcinoma"[ti] OR "asthma"[ti] OR "encephalitis"[ti] OR "diarrhoea"[ti] OR "diarrhea"[ti] OR "interferon"[ti] OR "postmenopausal women"[ti] OR "alemtuzumab"[ti] OR "granuloma"[ti] OR "sclerosis"[ti] OR "lymphocytic leukaemia"[ti] OR "lung cancer"[ti] OR "black smoke"[ti] OR "sulphate"[ti] OR "air pollution"[ti] OR "cardiovascular"[ti] OR "geriatric"[ti] OR "pertussis"[ti] OR "tetanus"[ti] OR "polio"[ti] OR "human papillomavirus vaccine"[ti] OR "HPV"[ti] OR "agranulocytosis"[ti] OR "haematopoietic"[ti] OR "malaria"[ti] OR "febrile illnesses"[ti] OR "corticotherapy"[ti] OR "JCV-specific"[ti] OR "psoriasis"[ti] OR "encephalopathy"[ti] OR "egg-allergic"[ti] OR "Serum C-reactive protein"[ti] OR "rheumatoid arthritis"[ti] OR "dengue fever"[ti] OR "cigarette smoking"[ti] OR "smoking"[ti] OR "leukoencephalopathy"[ti] OR "haemorrhagic"[ti] OR "hemorrhagic"[ti] OR "cirrhosis"[ti] OR "molluscum contagiosum"[ti] OR "organ transplant"[ti] OR " eczema"[ti] OR "hematopoietic cell transplantation"[ti] OR "melioidosis"[ti] OR "chronic hepatitis"[ti] OR "hepatitis"[ti] OR "peyronie's disease"[ti] OR "autism" OR "asthmatic" OR "pulmonary" OR "schizophrenia"[ti]) OR "nasal allergies"[ti]) OR "dental procedures"[ti]) OR "sedative medications"[ti]) OR "kidney injury"[ti]) OR "foetal bowel"[ti] OR "childbirth"[ti]) OR "brain-injured"[ti] OR "organ transplantation"[ti]) OR "cannabis"[ti]) OR "cocaine"[ti]) OR "pulmonary artery"[ti]) OR "rhinitis"[ti]) OR "tetanus"[ti]) OR "diphtheria"[ti]))) | 1,631,306 |
| **PubMed results** |  | ((#1 AND (#2 OR #3 OR #4 OR #5 OR #6)) AND #7) NOT #8  Filters: **Publication date from 1980/01/01; Humans; English** | 6966 |
| **Duplicates** |  | 47 articles removed | 6919 |
| **Screening of titles** |  |  | 6,575 |

**mh,** Mesh term; **ti,** title; **tiab,** title or abstract.

**Table S3.** Search strategy terms compiled using The Cochrane library (search timeline 1999-14 May 2014)

| **Category** | **Search number** | **Search terms** | **#items** |
| --- | --- | --- | --- |
| **Indication** | #1 | "influenza vaccines" or "influenza" or "influenza a" or "influenza b0" or "h1n1" or "h3n1" or "h5n1" or "haemophilus influenza" or "avian influenza" or "pandemic influenza" OR "influenza like illness" or "influenza-like illness" or "ILI" OR "influenza vaccine" or "influenza vaccine uptake" or "attenuated influenza" or "live attenuated influenza vaccine" or "hib" or "laiv" or "flumist" or "fluenz" or "influenza vaccinations" or "influenza epidemic" or "influenza epidemic outbreaks" or "influenza a/b" or "flu" or "flu vaccine" or "seasonal flu vaccine" or "pandemic influenza" or "seasonality" or "seasonal influenza epidemic" or "seasonal influenza vaccines" or "influenza pandemic" or "influenza immunisation" or "influenza immunization":ti,ab,kw | 4221 |
| **Models** | #2 | ("cost effectiveness" or "cost-effectiveness analysis" or "cost-effectiveness model" or "cost effectiveness model" or "cost-benefit model" or "cost-benefit analysis" or "cost burden" or "cost-burden" or "cost of illness" or "cost-analysis" or "cost analysis" or "dynamic transmission model" or "transmission model" or "dynamic transmission" or "effectiveness" or "economic modelling" or "economic decision modelling" or "decision model" or "economic evaluations" OR "quality of life" or "health related quality of life" or "HRQOL" or "QALY" or "quality adjusted life years" or "quality of health care" or "utility benefit" or "utility acceptability" or "utility cost" or "health impact":ti,ab,kw) OR "decision analyses" | 103,716 |
| **Spread of disease** | #3 | ("spread of disease" or "global burden" or "world health" or "infections" or "infectious" or "disease" or "prevention" or "control" or "treatment" or "pandemic" or "epidemic" or "risk factors" or "programmes" or "schedule" or "program" or "vaccination program" or "health policy" or "strategy" or "health" or "efficacy" or "dose" or "dosage" or "hospitalisation" or "hospitalization" or "respiratory infections" or "respiratory tract infections" or "pneumococcal" or "seasonality" or "respiratory" or "epidemic control" or "vaccine immunity" or "vaccination policy" or "natural immunity" or "vaccine effectiveness" or "duration of immunity" or "immunity timeline" or "annual production cycle" or "optimal target populations" or "transmissibility" or "epidemiological impact" or epidemiologic:ti,ab,kw) or (MeSH descriptor: [State Medicine] or [Immunity, Herd] or [Hospitalization) or ("pandemic" or "epidemic" or disease outbreak or "schedule" or strategy or "efficacy" or "disease control" or "disease control and prevention" or "control" or "disease prevention" or "centers for disease" or "centres for disease" or "vaccine uptake monitoring" or epidemic prevent or "referral" or "consultation" or "vaccine immunity" or "vaccine induced immunity" or "natural immunity" or "vaccine effectiveness" or "duration of immunity" or "immunity timeline") | 855,875 |
| **Reviews/reports** | #4 | “systematic review” or “target populations” or “systematic analysis” or “literature review” or “literature” or “department of health” or “public health” or “modelling study” or model or study or “mixing patterns” or “patterns” or “social contacts”:ti,ab,kw | 537,759 |
| **Mortality** | #5 | (“mortality” or “death” or “fatality”) or ( “mortality” or “death” or “cause of death” or “fatality” or “related death” or “related-death” or “fatal cases”:ti,ab,kw) | 62,176 |
| **Parameters** | #6 | “estimating” or “estimation” or “reproduction number” or “reproductive number” or “R0” OR “transmission parameters” or “mixing patterns” or “patterns” or “social contacts” :ti,ab,kw | 51,330 |
| **Target population** | #7 |  |  |
| **Exclusion criteria** | #8 | “pregnancy” or “diabetic” or “HIV” or “renal” or “diabetes” or “cancer” or “meningitis” or “methicillin-resistant Staphylococcus aureus” or “MRSA” or “Bifidobacterium” or “myocarditis” or “syncytial virus” or “pregnant” or “microbial exposure” or “brucellosis” or “splenectomy” or “heart disease” or “bronchiolitis” or “myeloid leukemia” or “carcinoma” or “asthma” or “encephalitis” or “diarrhoea” or “diarrhea” or “interferon” or “postmenopausal women” or “alemtuzumab” or “granuloma” or “sclerosis” or “lymphocytic leukaemia” or “lung cancer” or “black smoke” or “sulphate” or “air pollution” or “cardiovascular” or “geriatric” or “pertussis” or “tetanus” or “polio” or “human papillomavirus vaccine” or “HPV” or “agranulocytosis” or “haematopoietic” or “malaria” or “febrile illnesses” or “corticotherapy” or “JCV-specific” or “psoriasis” or “encephalopathy” or “egg-allergic” or “Serum C-reactive protein” or “rheumatoid arthritis” or “dengue fever” or “cigarette smoking” or “smoking” or “leukoencephalopathy” or “haemorrhagic” or “hemorrhagic” or “cirrhosis” or “molluscum contagiosum” or “organ transplant” or “ eczema” or “hematopoietic cell transplantation” or “melioidosis” or “chronic hepatitis” or “hepatitis” or “peyronie’s disease” or “autism” or “asthmatic” or “pulmonary” or “schizophrenia” or “nasal allergies” or “dental procedures” or “sedative medications” or “kidney injury” or “foetal bowel” or “childbirth” or “brain-injured” or “organ transplantation” or “cannabis” or “cocaine” or “pulmonary artery” or “rhinitis” or “tetanus” or “diphtheria”:ti,ab,kw | 298,127 |
| **Cochrane results** |  | ((#1 AND (#2 OR #3 OR #4 OR #5 OR #6)) AND #7) NOT #8 | 1749 |
| **Results available in The Cochrane Library** |  |  | 52 |
| **Duplicates** |  |  | 0 |
| **Screening of titles** |  | Titles not relevant to the objective of the study removed. | 21 |

**ab**, abstract; **kw**, keywords; **ti**, title.

**Table S4.** Search strategy terms compiled using the Google Scholar (conducted 7 May 2014 – 21 May 2014).

| **Category** | **Search number^a^** | **Search terms** | **#items** |
| --- | --- | --- | --- |
| **Indication,**  **Spread of disease,**  **Economic models,**  **Utility models,**  **Reviews/reports,**  **Numerical parameters,**  **Other parameters and**  **Mortality** |  | “Seasonality and influenza” or “Seasonality and influenza vaccine” or “seasonal influenza vaccine total doses” or “influenza and vaccination program” or “influenza vaccination program and target population” or “influenza vaccination” or “Influenza reproduction number” or “how to calculate R0” or “calculating standard deviation from standard error” or “planning influenza vaccination” or “estimation basic reproductive number and influenza” or “influenza and annual production cycle” or “age-specific global burden of disease” or “cost-burden pneumococcal disease” or “transmissibility and pandemic influenza” or “seasonal influenza and world health” or “vaccines recommended for adults” or “adult and adolescent immunisation health impact and cost effectiveness” or “expect and seasonal and pandemic influenza” or “interventions against influenza pandemic” or “burden of influenza and other respiratory infections” or “epidemiological impact and influenza vaccination” or “live attenuated vaccine and cost effectiveness” or “patient relevant clinical trial outcomes” or “hospital episode statistics” or “international population database” or “historical data graphs” or “influenza vaccine uptake” | 111 |
| **Duplicates compared to other search engines** |  | Only 1 duplicate found between PubMed and the Google Scholar. | 110 |

^a^ A combined search was conducted in the Google Scholar with each term(s) explored individually (i.e. “seasonality and influenza” is one term).

Supplementary Appendix 2: data for model adaptations with limited access to epidemiological and transmission data

Accompanying manuscript:

# “Cost-effectiveness analysis of the direct and indirect impact of intranasal live attenuated influenza vaccination strategies in children: alternative country profiles”

# Introduction

The core model framework has extensive capabilities to be adapted to alternative country profiles with limited access to epidemiological and transmission data. Model input parameters for the vaccine efficacy
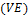
, natural immunity
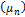
, vaccine induced immunity
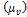
, and paediatric coverage with live attenuated influenza vaccine (LAIV) remain unchanged for all country profiles considered. For the mixing contact matrix
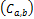
, in the absence of country specific data this was taken from Mossong et al.’s (26) European study, where demographics for Spain, Taiwan and Brazil were matched to those available in the study. Country specific data for the following were sourced from literature:

- Country demographics (population distribution, life expectancy, birth rates and mortality)
- Basic reproductive number
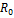
 (where possible otherwise base case value for England and Wales was applied)
- Incubation
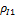
and infectious
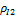
periods (where possible otherwise base value for England and Wales value was applied)
- Current vaccination policy (CVP) strategy for influenza vaccines
- Vaccination seasonal timelines
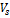

- Event rates
- Costs

## Model calculations

The model takes a compartmentalised approach which can be described by a series of partial differential equations. Each subpopulation is independently modelled using the same framework, as outlined below.

The initial population
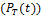
 is derived directly from the user input.

The effectively vaccinated population over time
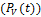
, is a sigmoidal approximation of the implementation of the user defined target vaccination strategy with peak vaccination following the pattern of influenza infection risk.

The susceptible (
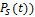
 population is derived from the initial population removing the effectively vaccinated population and those who become infected by cycle according to the equation:


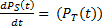
 -
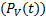
- ß.
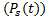


Where ß is the probability of exposure leading to infection. This (ß) is a sigmoidal function based itself on contact rate with infected individuals, peak probability of infection per contact and seasonality of influenza infection risk.

The exposed population
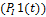
 is derived from the susceptible population according to the equation:


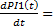
ß.
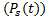
-α.
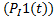


Where α is the transition probability to the infectious state. This is calculated as the inverse of the duration of latent infection per cycle.

The infectious population (
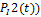
 is the product of transition from the latent state allowing for recovery and for the possibility of mortality linked to influenza infection, according to the equation:


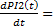
α.
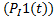
-(μ+ν)*
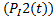


Where ν is the probability of recovery per cycle; the inverse of the duration of infection and μ the probability of mortality linked to influenza infection. It is assumed throughout the model that mortality due to other causes is zero.

For all population and large scale inputs where data exists, a normal distribution was modelled to the core data with > 99% fit in all cases.

In order to avoid irrational outputs caused by non-bounded inputs, irrational inputs (for example, negative population values) are not computed by the model

Alternative modelled distributions using the following showed similar results over 1000 iterations:

- Rates of ILI (Empirical distribution)
- Probabilities of healthcare related events e.g. hospitalisation, mortality (Beta and lognormal)
- Vaccine administration (Beta and triangular)
- Administration and other costs (Gamma)

Univariate sensitivity analyses vary one model parameter at a time to investigate the impact of the upper and lower limits. The univariate analyses consider the key model parameters. All sensitivity analyses consider (where available) a 95% confidence interval (CI) otherwise a 25% uncertainty margin is applied.

**Table S4.** Range of input values used for the univariate sensitivity analysis (tornado diagram).

| **Parameter** | **Mean** | **Range** |
| --- | --- | --- |
| Birth rate | 1.300% | 1.225-1.377% |
| Transmission probability for infection | 1.80 | 1.20-2.40 |
| QALY decrement per influenza event | 0.020 | 0.016-0.0243 |
| Incidence mortality per influenza event | 0.133% | 0.125-0.266% |
| Incidence primary care consultation | 5.960% | 4.891-7.029% |
| Incidence hospitalisation per influenza event | 0.150% | 0.125-0.175% |
| Cost per primary care consultation | £87.57 | £65.57-109.57 |
| Cost hospitalization | £2330.53 | £1687.53-2,974.00 |
| Cost vaccination - Admin. | £35.99 | £26.99-44.99 |
| Cost vaccination - TIV/QIV | £5.50 | £4.55-7.00 |
| Cost vaccination - LAIV | £14.00 | £10.00-18 |

# Country demographics

## Spain

Population data per age group, all-cause mortality rates, birth rates and life expectancy was taken from Instituto Nacional de Estadística (INE) (52, 81).

## Taiwan

The US Census population database provided the population data per age group and life expectancy (53). The Index Mundi online database provides the birth rates used in the model using reference year for 2012 (51). All-cause mortality taken from the US Census population and Wang et al. (2005) (65) to provide estimates over the elderly and non-elderly populations.

## Brazil

The US Census population database provided the population data per age group, life expectancy and mortality rates (53). The Index Mundi online database provides the birth rates used in the model using reference year for 2012 (51).

# Contact rates

Mossong et al.’s European study considered social contacts relevance to the spread of infectious disease –Belgium, Finland, Germany, Italy, Luxembourg, The Netherlands, Poland and Great Britain were included in the study (26). In the absence of country specific contact matrices for Spain, Taiwan and Brazil (although in Chowell et al. (50) homogenous mixing is assumed), demographics from these profiles were used to match to one of European countries considered in the study (26). Data for the following was used to identify the best match of mixing matrices:

- Average number of persons per room – taken from the United Nations (UN) compendium of human settlements statistics (27)
- Percentage of the population under the age of 15 years – taken from the UN’s department of economic and social affairs composition of the population (28)
- Percentage of the population under the age of 60 – taken from the UN’s department of economic and social affairs composition of the population (28)
- Percentage of population in urban setting – taken from the UN’s department of economic and social affairs world urbanisation prospects (29)
- Net enrolment ratios in secondary education – taken from the United Nations Educational, Scientific and Cultural Organisation (UNESCO) institute for statistics database for enrolment ratios into secondary education (30)
- Percentage employees – taken from the International Labour Office for the distribution of labour force by status in employment (ILO) (31)

Based on the information above, contact matrix for The Netherlands (26) was a suitable match for both Spain and Brazil, and Taiwan was matched to Poland.

# Infection and infective periods

The basic reproductive number
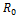
, incubation
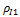
, and infectious
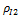
 periods for Spain and Taiwan was assumed the same as the base case,
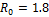
,
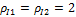
 days. For Brazil a mean value of
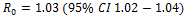
 taken from Chowell et al.(50) – based on a serial interval of 3 days of influenza defined by pneumonia and mortality time series in 27 Brazilian states. The value taken from (50) is low and may be reflective of the weak gradient in reproduction number with population size (particularly between north and south regions). The incubation
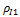
, and infectious
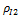
 periods for Brazil was also taken from (50), each lasting 1.5 days and a total infective period of 3 days.

# Current vaccination policies

## Spain

The CVP across all model age groups was based largely on information presented in Jimenez-Garcia et al. (77) – estimates of influenza vaccination across target population size of 17,860, 650 in Spain 2006-2007 season as per Spanish and ACIP recommendations is presented and supported by Jimenez-Garcia et al. (58) on coverage of universal vaccination in Spain (Madrid) for those aged 60 and above (reflected in the model CVP) and the Ministerio de Salud database (59). CVP coverage was estimated by taking proportions of high risk patients from each age group – with amendments using population demographic data to adjust where (58, 59, 77) were not in alignment to the model age bands. and assumptions applied to groups that were not age-defined (e.g. from (77) it was assumed that pregnant women, policemen and firemen fell into the 18-64 year old age group).

## Taiwan

In the absence of information for the paediatric population (model assumed 0% for these), estimates for CVP was taken from Chiu et al.(49) and supported by Wang et al.(54) - increase vaccination rate from 2000 to 2009. Wang et al.(54) considered the economic evaluation against influenza in the elderly (65 years and older) from a population-based influenza vaccination program in Taiwan, the study targeted 226,997 elderly people residing in Taipei county and reported an overall vaccination rate of 35.6% between 1 October and 31 December 2000. Chiu et al. investigated the influenza vaccination program for the elderly in Taiwan defined by those aged 65 years and older and reports a total vaccination rate of 43.77% in 2008-2009- which is the CVP rate considered in the model.

## Brazil

The CVP in Brazil was taken from the Ministerio de Saude database (51) using information from the national campaign against influenza vaccination 2014 26/04/2014 (vaccine coverage total Brazil).

# Seasonality and vaccination timelines

## Spain

The impact of influenza on hospital admissions during four flu season in Spain between 2000-2004 was reported by Lenglet et al. (63) –hospital discharge registers containing pneumonia, chronic bronchitis, heart failure and flu from all public hospitals for years 2000-2004; epidemic periods taken from the Sentinel Surveillance System and flu epidemics for seasons 2001/02, 2002/03 and 2003/04 (based on weekly hospitalisations per 100,000 in epidemic and non-epidemic periods) were presented. The influenza like illness (ILI) incidence rate per week across all epidemic periods was used to estimate the seasonality of influenza to reflect vaccination timelines in the model.

## Taiwan

Increased hospitalisation and fatal cases reported during the successive waves in the 2009 influenza pandemic in Taiwan 2010 was investigated by Yang et al.(64) to determine the possible contributors to the successive waves within this pandemic. Influenza surveillance data, and epidemic curves was used to conduct a large scale analysis of 4,703 isolates to monitor emergence, dominance and replacement of various variants. The monthly distribution of influenza isolates from the community outpatients, hospitalised patients confirmed by laboratory surveillance in Taiwan between May 2009 and April 2011 was used to populate the model vaccination timelines.

## Brazil

Alonso et al.(57) considered the seasonality of influenza in Brazil by modelling influenza related mortality and laboratory surveillance data. Monthly time series of pneumonia and influenza mortatlity from 1979-2001 across the 27 states was obtained – implying seasonality with mortality was greater in the southern states. Laboratory surveillance data of monthly influenza isolates between 2000-2005 supported claims that mortality peaks coincide with influenza virus activities. Averages over the laboratory surveillance data from (57) is applied to reflect vaccination timelines and seasonality of influenza in the model.

# Event rates

## Spain

Mortality and hospitalisation event rates based on values reported by Aballea et al. (56) for 50-64 year olds economic evaluation of influenza vaccination – consider case fatality rates (718 and 14 per 100,000 for high risk [HR] and low risk [LR] of complications) and hospitalisation among ILI cases seeking medical attention of 10% for HR and 1.3% for LR both based on synthesis of US data populate the model by taking averages over the HR and LR groups. The incidence of ILI consultations taken from the INE database (71) provide reference for the number of primary care consultations (PCC) in the model (8.13%).

## Taiwan

The distribution of influenza and pneumonia associated outpatient visits and hospitalisation in vaccinated and unvaccinated subjects from Chiu et al.(45) for 2008-2009 – provide model event rate estimates for PCC (vaccinated 5.57% and unvaccinated 4.82%) and hospitalisations (vaccinated 2.22% and unvaccinated 2.31%) by taking an average. Fatal influenza cases reported by Yang et al.(64) for influenza A and influenza B are used to provide model mortality events, by taking an average and adjusting for the number of events across the virus types.

## Brazil

Aballea et al. (58) provide the model event rates for mortality based on an average probability of death in high and low risk groups (HR: 0.826 and LR: 0.015-0.017); PCC taken from averaging % of GP visits for ILI in Brazil in HR (10%) and LR (5%); hospitalisations based on average the probability of events for HR (3.05) and LR (0.33).

# Resource use and costs

For all country profiles – the cost of LAIV was assumed to be the same referenced to £14.00 (42).

## Spain

Administrative costs of vaccines by a health worker is taken from De Juanes et al. (78) for the efficiency of influenza vaccination in the working population in Spain - €10.07 (2004). Cost of trivalent influenza vaccine (TIV) is based on Aballea et al. (56) - €3.83 (2007).

Hospitalisation cost for pneumonia and influenza events from Centre d’estudis en economia de la salud i de la polıtica social (SOIKOS) database (79) of €3,149 (2004) was considered. Statistical analysis of the basic minimum hospital data set from (80) for general practitioner (GP) costs for both standard and home visits is averaged €18.88 (2004) to provide a reference cost for PCC in the model. All costs were inflated to 2013 prices (52) and converted to GBP (67) for inclusion in the model.

## Taiwan

Vaccination cost, administration cost of vaccine and hospitalisation costs taken from Wang et al. (54). Direct costs for vaccination separated into drug cost $4.32 (2001), consultation $2.88 (2001) and administrative cost $0.34 (2001) is used to provide model estimates for TIV vaccination cost, PCC and administrative costs respectively. Hospitalisation cost of $3,304.91 (2001) - based on average cost per admission from (43) is considered in the model. All costs were inflated to 2014 US$ (68) and converted to GBP (67).

## Brazil

The international model by Aballea et al. (62) evaluates the cost-effectiveness of influenza vaccination for 50-64 year olds in Brazil, Germany, Italy and France and is used to obtain cost estimates for hospitalisation and PCC in the model. In the absence of cost estimates for vaccine administration the same cost for PCC is applied to the model. Standard GP visits from the societal perspective R$54 (2002) and hospitalisation costs for pneumonia and influenza related events of R$3, 653 (2002) are inflated to 2014 prices and converted to GBP (56). Cost of TIV was taken from the Governo Federal database (66) an estimate for the influenza vaccination is assumed to be £2.22 following conversion to GBP (67).

# Sensitivity analyses

Univariate sensitivity for each alternative country profile is given below (Fig. S1 –S3). For Spain the model is sensitive to cost of hospitalisation event, administration and the cost of LAIV. For Brazil, the model is sensitive to cost per vaccine (LAIV), QALY decrement per influenza event and cost of administration. The Taiwanese model is sensitive to the incidence of hospitalisation events, cost per vaccine (LAIV) and QALY decrement. The QALY decrement may be more impactful in the other profiles as they already include paediatric coverage in the CVP, whereas model estimates for Taiwan only consider the elderly population. The transmission of influenza in Taiwan may be reflective of the data inputted in the model where bias to the elderly population is possible.

## Spain


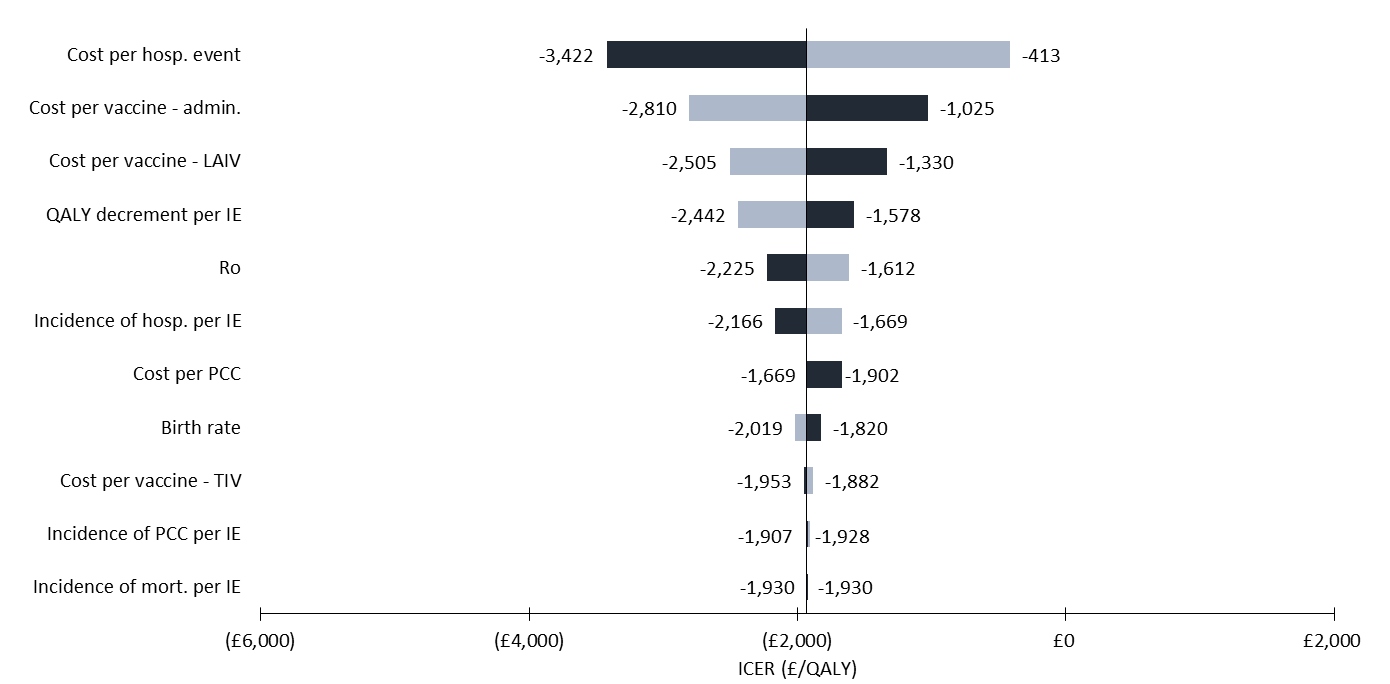


**Fig. S1.** Univariate sensitivity analysis for Spain in the ICER produced by applying a 95% confidence interval (CI) to the mean input parameter values. Admin., administration; Hosp., hospitalisation; ICER, incremental cost-effectiveness ratio; IE, influenza event; LAIV, live attenuated influenza vaccine; Mort., mortality; PCC, primary care consultation; QALY, quality adjusted life years; TIV, trivalent influenza vaccine; Vacc., vaccination.

## Taiwan


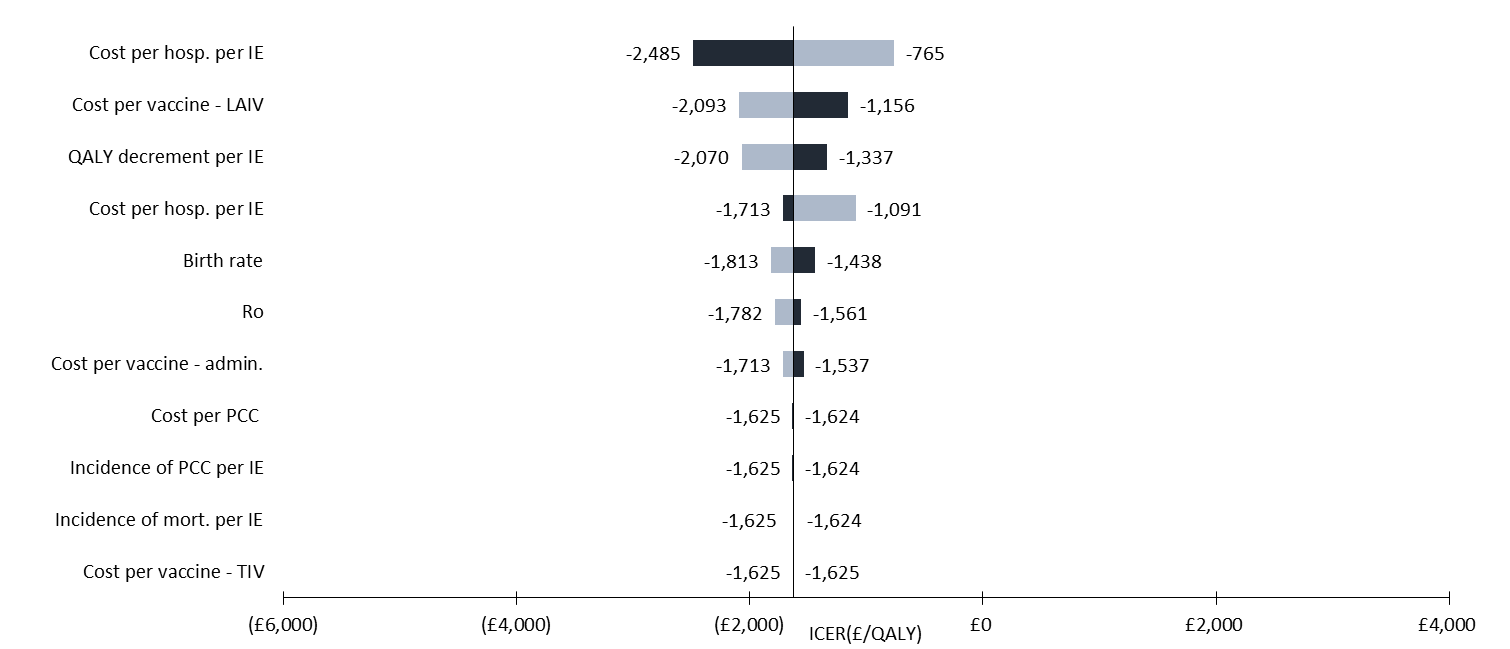


**Fig. S2. U**nivariate sensitivity analysis for Taiwan in the ICER produced by applying a 95% confidence interval (CI) to the mean input parameter values. Admin., administration; Hosp., hospitalisation; ICER, incremental cost-effectiveness ratio; IE, influenza event; LAIV, live attenuated influenza vaccine; Mort., mortality; PCC, primary care consultation; QALY, quality adjusted life years; TIV, trivalent influenza vaccine; Vacc., vaccination.

## Brazil


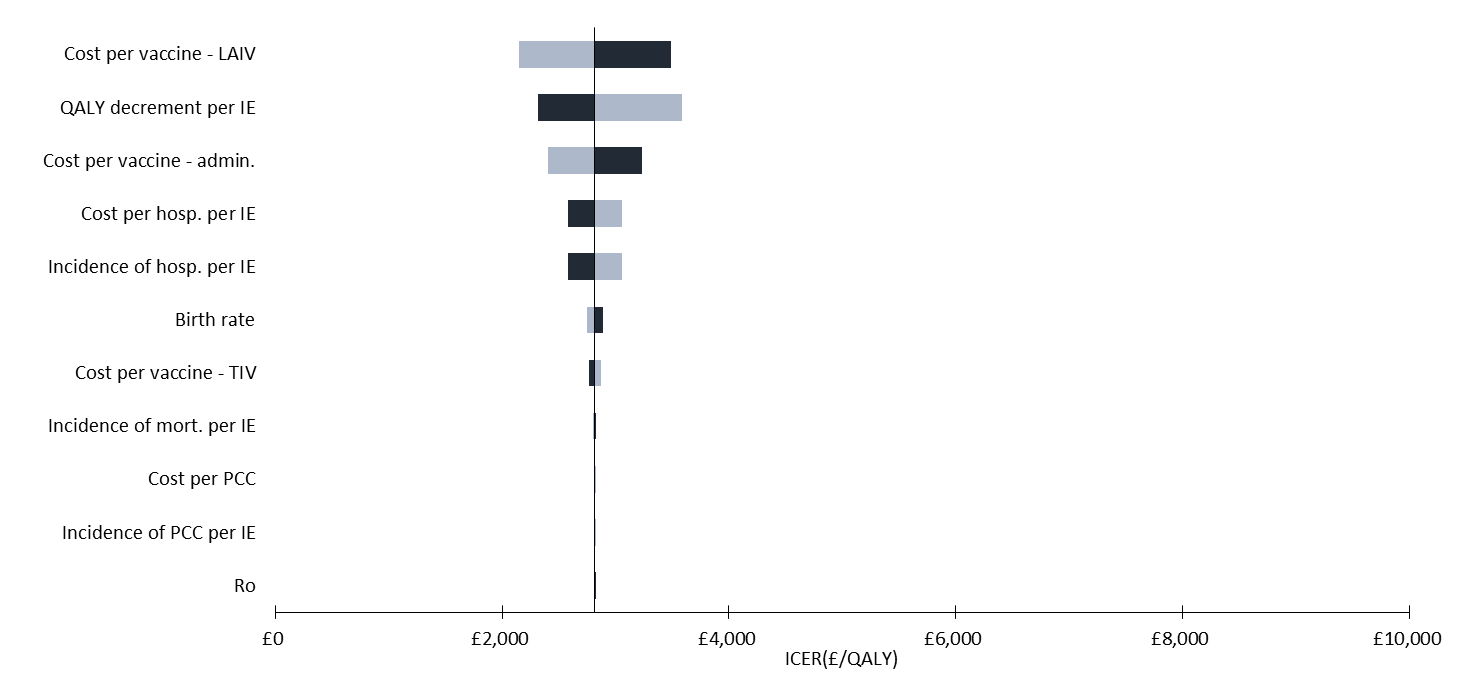


**Fig. S3. U**nivariate sensitivity analysis for Brazil in the ICER produced by applying a 95% confidence interval (CI) to the mean input parameter values. Admin., administration; Hosp., hospitalisation; ICER, incremental cost-effectiveness ratio; IE, influenza event; LAIV, live attenuated influenza vaccine; Mort., mortality; PCC, primary care consultation; QALY, quality adjusted life years; TIV, trivalent influenza vaccine; Vacc., vaccination.
